# Multiple Imaging Modalities in the Diagnosis of Infective Endocarditis

**DOI:** 10.31083/RCM39571

**Published:** 2025-09-25

**Authors:** Hanwen Zhang, Jimin Zhang, Jingjin Wang, Haiping Wang, Changming Xiong

**Affiliations:** ^1^Respiratory and Pulmonary Vascular Center, State Key Laboratory of Cardiovascular Disease, Fuwai Hospital, National Center for Cardiovascular Diseases, Chinese Academy of Medical Sciences and Peking Union Medical College, 100037 Beijing, China; ^2^4+4 Medical Doctor Program, Chinese Academy of Medical Sciences & Peking Union Medical College, 100037 Beijing, China; ^3^Department of Echocardiography, State Key Laboratory of Cardiovascular Disease, Fuwai Hospital, National Center for Cardiovascular Diseases, Chinese Academy of Medical Sciences and Peking Union Medical College, 100037 Beijing, China; ^4^Department of Radiology, Fuwai Hospital, National Center for Cardiovascular Diseases, Chinese Academy of Medical Sciences and Peking Union Medical College, 100037 Beijing, China

**Keywords:** infective endocarditis, multimodal imaging, echocardiography, nuclear imaging, magnetic resonance imaging

## Abstract

Infective endocarditis (IE) is an inflammatory disease caused by the infection of the endocardium or heart valves by pathogenic microorganisms. It is characterized by diagnostic challenges, difficult treatment, and high mortality. Multimodal imaging techniques, including echocardiography, computed tomography (CT), magnetic resonance imaging (MRI), and nuclear medicine imaging, play a crucial role in the diagnosis of IE. Echocardiography is the first-line imaging modality for suspected IE. Cardiac CT, with its excellent spatial resolution and three-dimensional (3D) reconstruction capabilities, is helpful in detecting paravalvular abscesses, fistulas, and pseudoaneurysms. MRI has advantages in identifying neurological complications and assessing myocardial involvement. Nuclear imaging demonstrates high specificity in detecting prosthetic valve IE and device-related infections. These imaging techniques are important in detecting perivalvular complications, evaluating local and distant spread of infection, and guiding therapeutic interventions, thereby enhancing the diagnostic and therapeutic management of IE.

## 1. Introduction

Infective endocarditis (IE) is a severe and potentially life-threatening disease characterized by the infection of cardiac valves and involvement of multiorgan systems. Although therapeutic strategies have improved, the overall mortality remains high. Data from the 2019 Global Burden of Disease Study indicate that IE has an estimated global incidence of 13.8 cases per 100,000 patient-years, with a marked gender disparity—610,000 cases reported in males versus 480,000 in females. In-hospital mortality persists at approximately 20%, accounting for 66,300 global deaths in 2019, a 131% increase since 1990 [1, 2, 3]. A prompt and precise diagnosis is critical for initiating therapy and enhancing long-term prognosis.

**Fig. 1.**
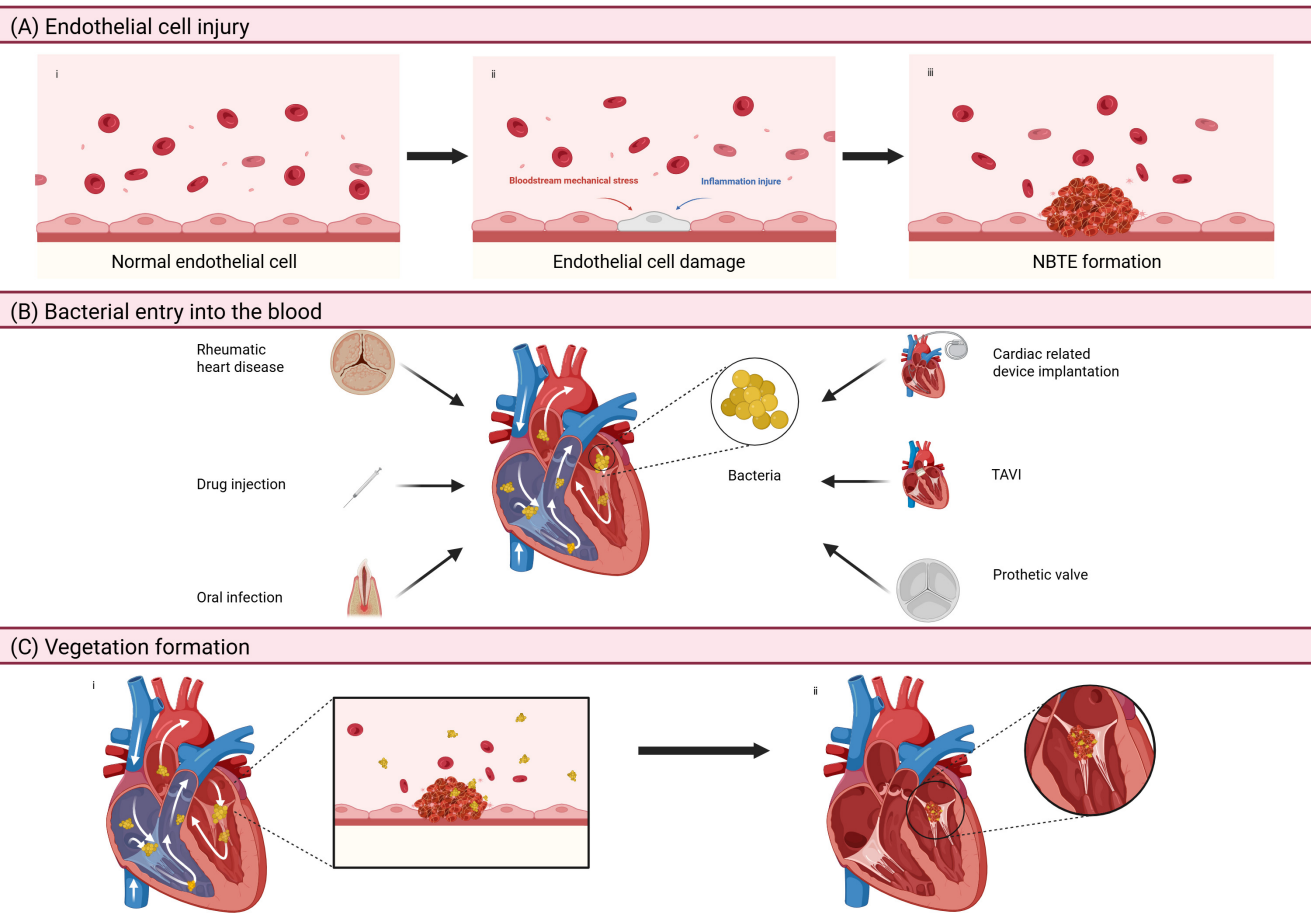
**The pathogenesis of IE**. (A) Endothelial cell injury: mechanical damage and inflammation can lead to injury of the endothelial tissue, causing exposure to the extracellular matrix under the endothelium, which activates platelets and leads to the formation of NBTE. (B) Bacterial entry into the blood: rheumatic heart disease, drug injection, oral infection, cardiac related device implantation, TAVI, and prosthetic valve are all reasons for the entry of bacteria into heart through the circulatory system. (C) Vegetation formation: parts of the protein on the bacteria’s surface can interact with platelets and endothelial cells, contributing to the colonization of bacteria in the endocardium and non-bacterial thrombosis. IE, infective endocarditis; NBTE, non-bacterial thrombotic endocarditis; TAVI, transcatheter aortic valve implantation. Figure created with BioRender.

The pathogenesis and complications of IE stem from the intricate interactions among pathogenic microorganisms, valve endothelium, and host immune responses. Mechanical injury combined with inflammation usually causes endothelial injury, leading to the exposure of the subendothelial extracellular matrix. This pathological change triggers platelet activation and the subsequent formation of fibrin-platelet aggregates, a phenomenon clinically known as non-bacterial thrombotic endocarditis (NBTE) [4]. During the onset of bacteremia, circulating pathogens adhere to cardiac structures through surface protein-mediated interactions with platelets and endothelial cells, promoting the colonization of microorganisms on the endocardium surfaces and the progression of thrombotic lesions [5] (Fig. 1). Invasive infections stimulate inflammatory cell infiltration, cytokine cascade reactions, and the release of coagulation factors. These collective mechanisms promote the formation of vegetations, which result in irreversible valvular destruction. During the acute phase of IE, embolic vegetations may obstruct vascular beds, especially in cerebral and pulmonary circulation.

**Fig. 2.**
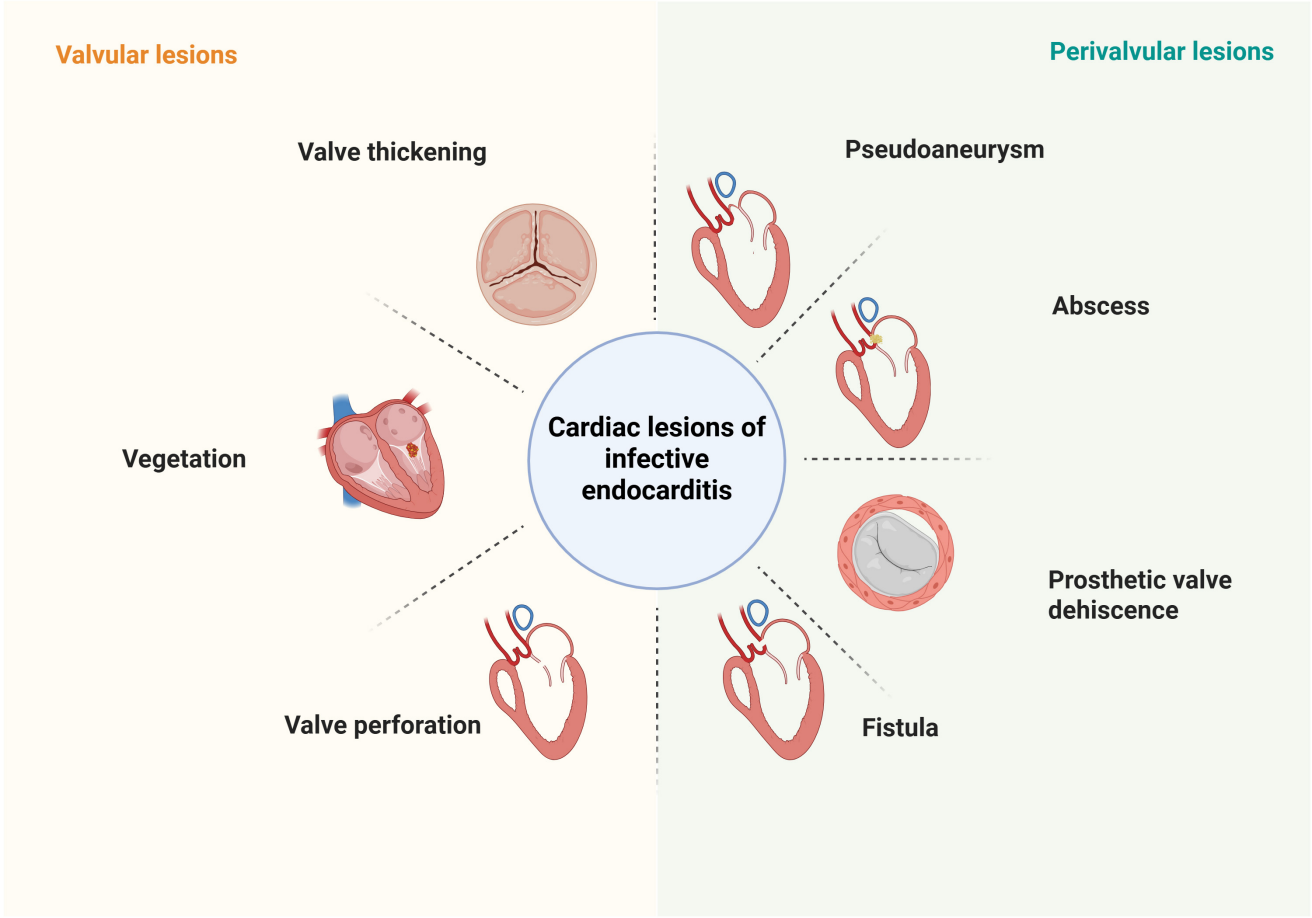
**Characteristics of different cardiac lesions of IE detected by imaging modalities**. Valvular lesions include valve thickening, vegetation, and valve perforation; Perivalvular lesions include pseudoaneurysm, abscess, prosthetic valve dehiscence, and fistula. IE, infective endocarditis. Figure created with BioRender.

According to the 2023 European Society of Cardiology (ESC) Guidelines and the updated modified Duke criteria, blood culture analysis and multimodality imaging are the cornerstone diagnostic modalities for IE (Fig. 2). Among the major criteria, the 2023 ESC guidelines have added the definition of “imaging positive”. That is, the anatomical and metabolic lesion characteristics of valves, perivalvular/periprostheses in IE detected by any one of the imaging techniques such as echocardiography, cardiac computed tomography (CT), ^18^F-fluorodeoxyglucose positron emission tomography ([^18^F]-FDG-PET/CT) and white blood cell single photon emission tomography/computed tomography (WBC SPECT/CT). However, blood cultures have a significant false-negative rate, particularly in patients who have received prior antibiotic therapy. Advanced imaging techniques play a pivotal role in confirming the diagnosis of IE, especially in suspected cases with negative cultures. This review studies the latest advancements in the applications of multimodality imaging for the evaluation of IE (Table 1, Ref. [6, 7, 8, 9, 10, 11, 12]).

**Table 1.** **The advantages and disadvantages of different imaging modalities in diagnosing IE**.

	Advantage	Disadvantage	Sensitivity	Specificity	Clinical scenarios where preferred
Echocardiography	Real-time assessment of cardiac structures, blood flow, and function	TTE is limited for detecting small vegetations (<5 mm)	71% (TTE for NVE) [6];	80% (TTE for NVE) [6];	TTE for first-line imaging modality
			96% (TEE for IE) [7]	83% (TEE for IE) [7]	
	Satisfactory diagnostic accuracy of vegetations (TEE > TTE)	Limited for detecting perivalvular complications, especially for PVE	65% (TTE for PVE) [8];	86% (TTE for PVE) [9];	TEE for patients with high clinical suspicion for IE and a negative or inadequate TTE
			91% (TEE for PVE) [8]	94% (TEE for PVE) [9]	
	Satisfactory diagnostic accuracy of perivalvular complications (TEE > TTE)	High dependence on the operator’s experience	57% (TTE for CIED-IE) [10];	18% (TTE for CIED-IE) [10];	
			61% (TEE for CIED-IE) [10]	47% (TEE for CIED-IE) [10]	
	Convenient operation				
	No radiation				
CT	High diagnostic accuracy of perivalvular complications	Limited for detecting small vegetations (<10 mm)	85.7% (IE) [7]	83.8% (IE) [7]	CT for the detection of valvular lesions and paravalvular complications in cases of inconclusive echocardiography
	CTA may replace coronary angiography in preoperative patients who plan to undergo endocarditis surgery	Risk of nephrotoxicity	78% (PVE) [7]	94% (PVE) [7]	
	Extracardiac imaging scenarios are accessible, such as embolisms (hemorrhagic cerebral complications) and abscesses (splenic, renal and hepatic abscesses)	Radiation exposure			
MRI	High diagnostic accuracy of small neurological lesions	Low spatial resolution	NA	NA	MRI for the screening of peripheral lesions (central nervous system, myocarditis) in IE patients
	Detection of valvular vegetation and extension of inflammation	Constrained by signal voids created by prostheses			
	Functional evaluation of valve and ventricle				
	No radiation				
^18^F-FDG PET/CT	Applicable for detecting intracardiac infection	Limited for the detection of NVE	31% (NVE) [8]	98% (NVE) [8]	^1^^8^F-FDG PET/CT for detecting infectious lesions in cases of inconclusive echocardiography or culture-negative IE, possible PVE and possible CIED-IE
	Useful in assessing extracardiac complications	Longer time scanning	86% (PVE) [8]	84% (PVE) [8]	
	High sensitivity in diagnosing PVE and CIED-IE		72% (CIED-IE) [8]	83% (CIED-IE) [8]	
^99m^Tc-HMPAO SPECT/CT	High specificity in diagnosing PVE and CIED-IE	Longer time scanning	86% (IE) [11]	97% (IE) [11]	^99m^Tc-HMPAO SPECT/CT is used when echocardiography is negative, or inconclusive and PET/CT is not available in patients with high clinical suspicion of PVE
	Useful in assessing extracardiac infection	Lower spatial resolution compared to ^18^F-FDG PET/CT	64% (PVE) [10]	100% (PVE) [10]	
		Limited in detecting smaller vegetation	84% (CIED-IE) [12]	88% (CIEsD-IE) [12]	
		Requires blood handling			

^18^F-FDG PET/CT, ^18^F-fluorodeoxyglucose positron emission tomography/computed; ^99m^Tc-HMPAO SPECT/CT, single-photon emission tomography and computed tomography with technetium^99m^-hexamethylpropyleneamine oxime-labelled leucocytes; CIED-IE, cardiac implantable electronic device-related infective endocarditis; CT, computed tomography; CTA, computed tomography angiography; IE, infective endocarditis; MRI, magnetic resonance imaging; NVE, native valve endocarditis; PVE, prosthetic valve endocarditis; TEE, transesophageal echocardiography.

## 2. Native Valve Endocarditis

Native valve endocarditis (NVE) is relatively rare, with an estimated incidence of approximately 2–10 cases per 100,000 person-years [13]. Although it was traditionally linked to rheumatic heart disease, more recent epidemiological data highlight degenerative valve disease as the primary risk factor, especially in aging populations. Younger patients are more likely to be found in low-resource settings. In more developed regions, earlier interventions for congenital heart disease (e.g., catheter-based therapies) and intravenous drug use have led to a growing number of cases in adolescents and young adults.

### 2.1 Echocardiography

Echocardiography, including both transthoracic echocardiogarphy (TTE) and transesophageal echocardiography (TEE), is the first-line imaging modality in suspected IE [14]. Due to its excellent clinical applicability, non-invasiveness, and high diagnostic capability, this technique can comprehensively assess the severity of the disease and the risk of embolism. A systematic echocardiographic examination can fully analyze the characteristics of vegetations (size, shape, mobility, and anatomical distribution), identify perivalvular complications (abscesses, pseudoaneurysms, or prosthetic valve dehiscence), and detect structural abnormalities including intracardiac fistulas and valvular perforations, thereby providing essential crucial morphological and functional information for treatment decisions.

Early diagnosis of IE through the identification of vegetations by echocardiography is of great importance. Studies have shown that the sensitivity and specificity of TEE are 70% and 80% respectively, while those of TEE exceed 85% accuracy in both aspects [6]. The main reason for the limited sensitivity of TTE primarily lies in its inability to effectively detect smaller vegetations (<5 mm). The sensitivity of TTE in detecting small vegetations (<5 mm) and large vegetations (6–10 mm) is 25% and 90%, respectively [15]. TTE performs poorly in identifying perivalvular lesions, while TEE has higher sensitivity and can compensate for the deficiency of TTE. A meta-analysis has shown that the sensitivity, specificity, positive predictive value (PPV), and negative predictive value (NPV) of TEE are 97%, 88%, 97%, and 88% respectively [16]. Although TEE has a higher sensitivity, TTE is still recommended as the preferred imaging modality for the assessment of suspected NVE. When the initial TTE results are inconclusive or negative but IE is highly suspected clinically, TEE should be performed immediately. TEE provides higher spatial resolution through posterior cardiac window imaging, which is especially helpful in detecting vegetations with diameters of less than 5 mm. Heart failure is one of the most common complications of IE, affecting approximately 14% of patients, and is the main cause of valve dysfunction [17]. A chest X-ray examination can rule out lung diseases with manifestations similar to heart failure, while TTE remains the gold standard for confirming structural abnormalities of the heart. Common manifestations include decreased ejection fraction, ventricular dilation, and valve regurgitation due to valve perforation [18].

Although echocardiography plays a key role in the diagnostic assessment of IE, it also has certain limitations, including the high dependence on operators’ experience and significant differences in accurately detecting pathology. In the detection of small vegetations, perivalvular abscesses, and pseudoaneurysms, the sensitivity of TTE is significantly lower than that of advanced imaging techniques. Therefore, in cases with atypical clinical manifestations, other imaging techniques are often needed for diagnostic confirmation [7]. Echocardiography techniques should be selected based on individual patient conditions and integrated with other imaging examinations to improve the overall diagnostic accuracy of complex endocarditis cases.

### 2.2 Cardiac Computed Tomography 

**Fig. 3.**
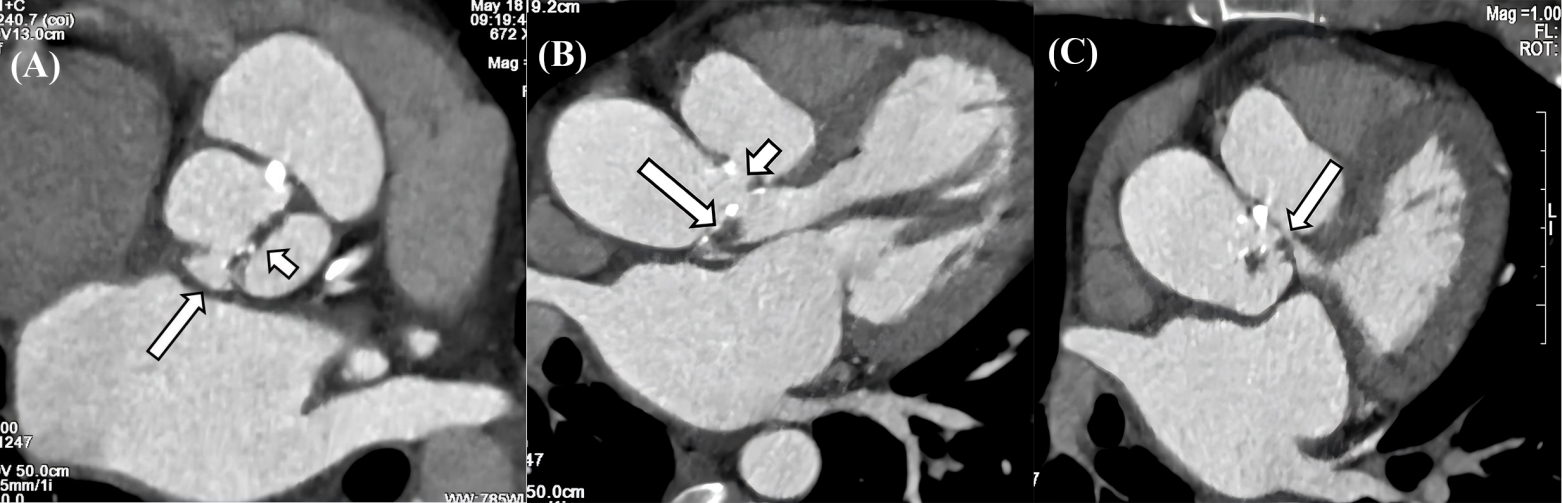
**CT characteristics of IE**. (A) Valve thickening, calcification and penetrating ulcer in the aortic valve. The aortic sinus consists of two sinuses, and the aortic valve is a bicuspid valve. There is thickening and calcification of the valve leaflets (long arrow), and a penetrating ulcer is formed in the right coronary sinus (short arrow). (B) Vegetation, pseudoaneurysm, and leakage in aortic valve. The aortic valve leaflets show thickening with vegetations formation (long arrow), and a pseudoaneurysm is observed on the aortic valve, along with a large leakage between the aorta and the valve (short arrow). (C) Pseudoaneurysm with LVOT communication. A large pouch-like structure is present in the left anterior aspect of the aortic sinus, situated above the aortic valve, with small channels observed communicating with the LVOT (long arrow). CT, computed tomography; IE, infective endocarditis; LVOT, left ventricular outflow tract.

Cardiac computed tomography (CCT) is an important diagnostic tool in the management of IE, due to its high spatial resolution (slice thickness ≤0.5 mm) and advanced three-dimensional (3D) multiplanar reconstruction to precisely delineate perivalvular anatomy. This technique provides superior sensitivity regarding the detection of perivalvular abscesses, fistulous tracts, and pseudoaneurysms, which often evade conventional echocardiographic techniques [19]. A recent study shows that CT detected pseudoaneurysms in 14% of IE patients, while echocardiography only detected them in 4% of IE patients [20] (Fig. 3).

**Fig. 4.**
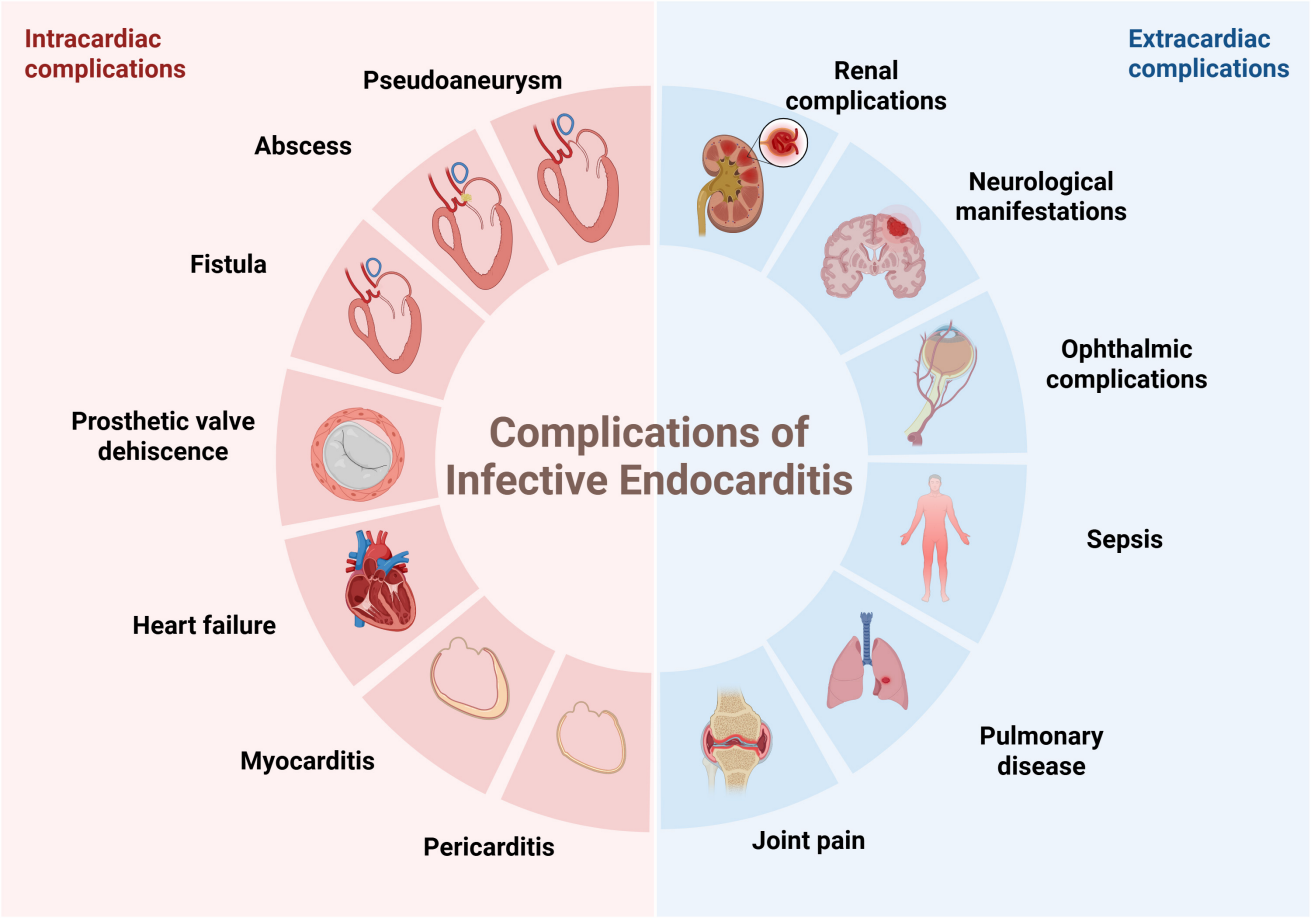
**The intracardiac and extracardiac complications of infective endocarditis**. Figure created with BioRender.

In patients with NVE, when TTE or TEE is contraindicated or yields inconclusive results, CCT can serve as an important complementary diagnostic tool. A recent study revealed that CCT exhibits moderately higher diagnostic accuracy than TTE and TEE, with rates of 85.7%, 50.6% and 59%, respectively. Ultimately, all three imaging modalities together improved the diagnostic accuracy by 87.9% [7, 21, 22]. Cardiac computed tomography angiography (CTA) may be used to replace invasive catheter-guided coronary angiography in patients who undergo surgery, providing a non-invasive assessment of the coronary anatomy and avoiding the risk of vegetation embolization [23]. In addition, IE often leads to extracardiac complications, mainly caused by infectious embolism or systemic inflammatory responses. Common affected sites include the central nervous system (CNS), kidneys, lungs, joints, and eyes (Fig. 4). CCT can often simultaneously detect these lesions, providing important clues for the diagnosis of IE [24]. This comprehensive imaging approach is especially helpful in challenging clinical scenarios, as it enables systematic evaluation of dominant infectious foci and has important consequences for therapeutic decisions [25].

The 2023 ESC and Duke-International Society of Cardiovascular Infectious Diseases (ISCVID) guidelines have incorporated CCT as a major imaging modality for IE. However, a number of important limitations must be acknowledged. Low temporal resolution of CCT restricts the detection of vegetations smaller than 10 mm and the assessment of functional valvular competence [26]. Additional limitations include radiation exposure and the risk of contrast-related nephrotoxicity, which are of particular concern in patients with impaired renal function resulting from low cardiac output, glomerulonephritis, or the use of nephrotoxic antibiotics. Although these limitations exist, CCT continues to play a vital role in assessing complications associated with IE, and it is a helpful tool when used with caution after considering potential risks and benefits.

### 2.3 Magnetic Resonance Image 

Cardiac magnetic resonance imaging (CMR), with its high spatial resolution and multi-parameter imaging capabilities, can comprehensively assess the characteristics of myocardial tissue. This technique can identify inflammatory congestion or edema through T2-weighted imaging, detect necrosis and fibrosis through late gadolinium enhancement (LGE), and evaluate pericardial thickening (>4 mm) and its inflammatory activity (high signal on T2-STIR) [27]. In the detection of myocarditis and pericarditis, CMR may exhibit characteristic manifestations including myocardial edema, late gadolinium enhancement and pericardial effusion. Despite its superior spatial resolution, CMR has a lower temporal resolution than echocardiography and often fails to capture smaller vegetations. Therefore, the 2023 ESC guidelines and the 2023 Duke-ISCVID IE diagnostic criteria did not include MRI as a primary diagnostic criterion [26, 28].

MRI is regarded as the gold standard for detecting neurological complications of IE, and it is more sensitive than CT for detecting neurological lesions, found in 60–80% of IE cases [29]. A meta-analysis of the diagnostic efficacy of brain MRI in 2133 patients with suspected or confirmed IE revealed that the detection rates of acute ischemic lesions, cerebral microbleeds, hemorrhagic lesions, abscesses or meningitis, and intracranial mycotic aneurysms were 61.9%, 52.9%, 24.7%, 9.5% and 6.2%, respectively [30]. In a single-center study, Papadimitriou-Olivgeris *et al*. [31] found that 42% patients suspected of IE who underwent brain imaging had neurological lesions. However, brain MRI demonstrated limited ability to modify the diagnosis from rejected to possible or from possible to definite, with 0% and 2% in asymptomatic patients with IE suspicion (1% and 4% in symptomatic patients) [31]. A retrospective study reached a similar conclusion, indicating that routine MRI examination was unable to reduce mortality within 3 months in asymptomatic left-sided IE patients [32]. Although the role of MRI in the final diagnosis of IE is limited, its influence on treatment decisions is more significant. Cerebral embolic events detected by MRI, result in a change in the indication for surgery in about 20% of patients [31], thereby helping to improve the prognosis and reduce 6-month mortality [33]. In conclusion, MRI has unique advantages in simultaneously evaluating complications of both the central nervous system (stroke, cerebral abscesses) and cardiovascular system (myocarditis, pericarditis), which contributes to its role as an important imaging modality for comprehensive IE assessment, particularly in complex cases requiring multiorgan evaluation.

### 2.4 Nuclear Imaging Techniques

#### 2.4.1 ^18^F-FDG PET/CT 

^18^F-FDG PET/CT is a molecular imaging modality that uses the radioactive ^18^F-FDG to detect hypermetabolic inflammatory lesions. This technique is based on the mechanism of increased expression of glucose transporter proteins (GLUTs) and elevated hexokinase (HXK) enzymatic activity in activated immune cells (particularly macrophages and lymphocytes), which exhibit upregulated glycolytic metabolism during inflammatory responses. Following its uptake, intracellular ^18^F-FDG generates quantifiable PET signals, thereby enabling precise localization of infective/inflammatory lesions by mimicking the uptake patterns of glucose [34].

This imaging technique has high specificity but moderate sensitivity, thereby serving as a complementary diagnostic method to echocardiography and CT scans, compensating for their limitations in spatial resolution. In a recent meta-analysis of 26 studies involving 1358 patients, the sensitivity and specificity of ^18^F-FDG PET/CT for NVE were 31% and 98%, respectively [8]. Its ability to distinguish NVE from other diseases is limited, with an area under the receiver operating characteristic (ROC) curve of 0.609 for NVE [35]. However, if ^18^F-FDG PET/CT indicates evidence of intracardiac infection, it has high predictive value for diagnosis, especially when other examination methods such as echocardiography cannot provide definitive results.

^18^F-FDG-PET/CT cannot only evaluate intracardiac lesions but can also simultaneously detect extracardiac complications such as mycotic aneurysms and septic emboli [36]. In patients suspected of NVE, this technique identifies systemic embolic events in 42% of cases, thereby enhancing its diagnostic accuracy compared with echocardiography alone [35]. By reclassifying certain cases as “definite IE”, ^18^F-FDG-PET/CT supports clinicians in making decisions and accelerating the initiation of treatment, thus improving patient prognosis [37]. A multimodal imaging approach, integrating echocardiography, CT, MRI, and targeted organ-specific techniques, enables comprehensive evaluation of both intracardiac and extracardiac IE-related complications (Fig. 3).

Nevertheless, the diagnosis of IE remains challenging in the presence of co-infections, other inflammatory diseases, or malignancies. As a result, a qualitative scoring system with semi-quantitative methods to assess hypermetabolism of the spleen or bone marrow (HSBM) has been proposed to enhance the suspicion of IE. This scoring system consists of a four-point score model (0 = no focal cardiac uptake; 1 = focal cardiac uptake < blood-pool activity; 2 = blood-pool < focal cardiac uptake < liver activity; 3 = focal cardiac uptake > liver), and is combined with HBSM (bone marrow or spleen-to-liver ratio >1) [38]. Philip *et al*. [39] proposed that diffuse splenic uptake in ^18^F-FDG PET/CT may become a novel diagnostic indicator for NVE, with a specificity of 83.3%. The diagnostic efficacy of ^18^F-FDG PET/CT is constrained by its relatively low spatial resolution (approximately 5 mm), which restricts its ability to detect smaller lesions. False-negative results may also occur in patients with prior antibiotic exposure [40], as well as in scenarios of attenuated inflammatory responses or biofilm formation—the latter causing reduced metabolic activity and subsequent diminished ^18^F-FDG detection accuracy.

#### 2.4.2 ^99m^Tc-HMPAO-SPECT/CT 

Similar to ^18^F-FDG PET/CT, single-photon emission tomography and computed tomography with technetium^99m^-hexamethylpropyleneamine oxime-labelled leucocytes (^99m^Tc-HMPAO-SPECT/CT), also known as WBC SPECT/CT, is a hybrid molecular imaging modality that integrates SPECT and CT. This technique labels autologous leukocytes using ^99m^Tc-HMPAO and takes advantage of its natural chemotactic property of migrating to infectious/inflammatory lesions. The synergistic combination of SPECT 3D tomographic imaging and CT anatomic co-registration enables precise localization of IE vegetations and metastatic abscesses. This modality has functional imaging capability at the molecular level and can conduct a comprehensive assessment of multi-organ involvement throughout the body in a single examination. Its unique strength lies in early IE detection, and is especially advantageous in early diagnosis [41].

^99m^Tc-HMPAO-SPECT/CT demonstrates a superior performance in the diagnosis of IE, with a sensitivity of 90% and a specificity of 100% [42]. In a head-to-head comparison study between TTE and ^99m^Tc-HMPAO-SPECT/CT (N = 40), the latter demonstrated better diagnostic ability: PPV improved from 46% in TTE to 81% in SPECT/CT, and the specificity increased from 42% to 88%. Most importantly, this modality provides additional diagnostic value under the modified Duke criteria. By identifying latent paravalvular uptake lesions, it reclassified 27% of misclassified “possible IE”, thereby optimizing therapeutic decision-making [43].

However, this method also has limitations. ^99m^Tc-HMPAO-SPECT/CT involves longer scanning times and requires multiple imaging acquisitions. Furthermore, its imaging quality may be affected by factors such as neutropenia and smaller lesions, and its spatial resolution is lower than that of ^18^F-FDG PET/CT [44]. Among patients receiving antibiotic treatment, the false-negative rate may increase, decreasing the diagnostic accuracy and possibly misdiagnosing non-IE patients [45]. Thus, ^99m^Tc-HMPAO-SPECT/CT is currently mainly used as a supplementary examination method and further research is needed to verify its clinical value.

## 3. Prosthetic Valve Endocarditis

Prosthetic valve endocarditis (PVE), refers to the invasion of prosthetic heart valves or surgically reconstructed native valves by infectious miccroorganisms, accounting for 20% to 30% of all cases of IE. Among patients undergoing prosthetic valve replacement surgery, the incidence rate is 1%–6%, equivalent to 0.3%–1.2% per patient-year. PVE can be classified into early (within 12 months post-implantation) and late (more than 12 months) phases. Early PVE is predominantly caused by perioperative contamination, often involving the sewing ring and annulus, accompanied by complications such as paravalvular abscesses, prosthesis dehiscence, and pseudoaneurysms. In contrast, late PVE is pathophysiologically similar to NVE. The pathogen spreads through the bloodstream to the high-flow-velocity areas of the valve. Although the onset is later, it can cause similar tissue destruction [26]. Due to the difficulty in diagnosis and complexity of therapeutic interventions, the prognosis of PVE is usually poor.

**Fig. 5.**
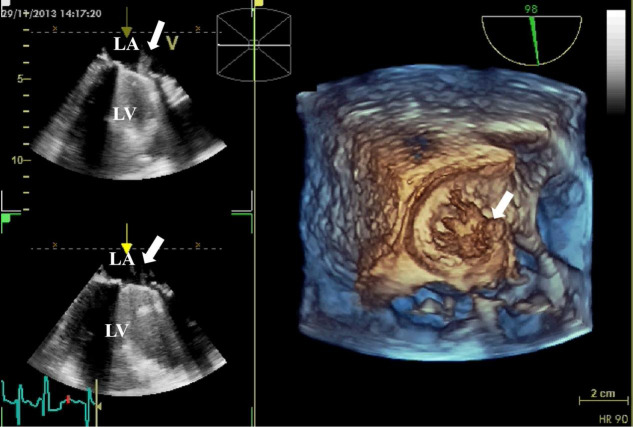
**3D TEE detection of mechanical valve vegetation**. 3D TEE indicates the formation of vegetation (white arrow) on the mechanical valve of the mitral valve. (Left) The 2D views show vegetation on the surface of the mechanical valve. (Right) The 3D view shows the formation of vegetation on the mechanical valve. 3D, three-dimensional; 2D, two-dimensional; TEE, transesophageal echocardiography; LA, left atrium; LV, left ventricle.

**Fig. 6.**
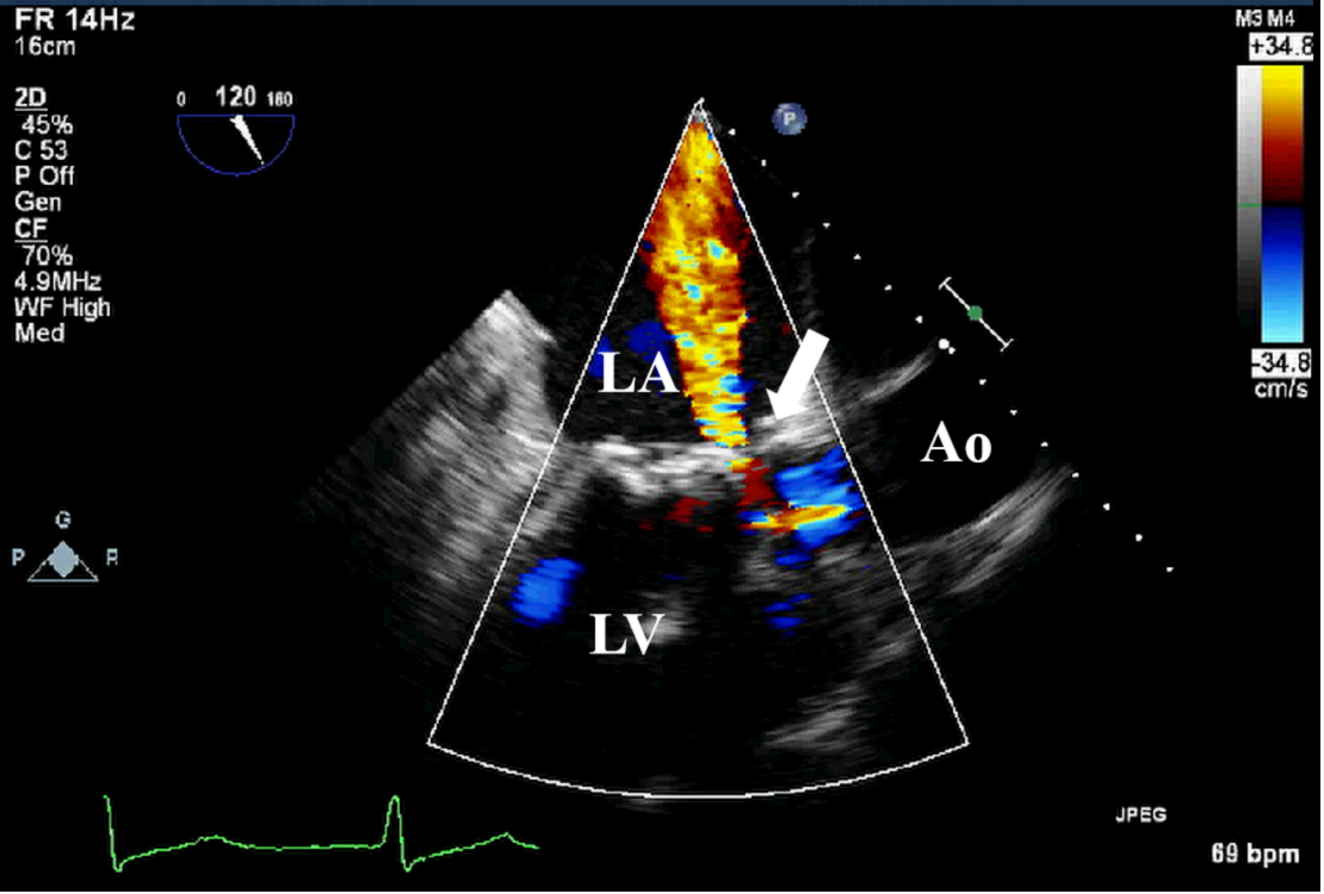
**TEE detection of bioprosthetic valve paravalvular leak**. TEE shows paravalvular leakage (white arrow) of bioprosthetic mitral valve in mid-esophageal view. TEE, transesophageal echocardiography.

**Fig. 7.**
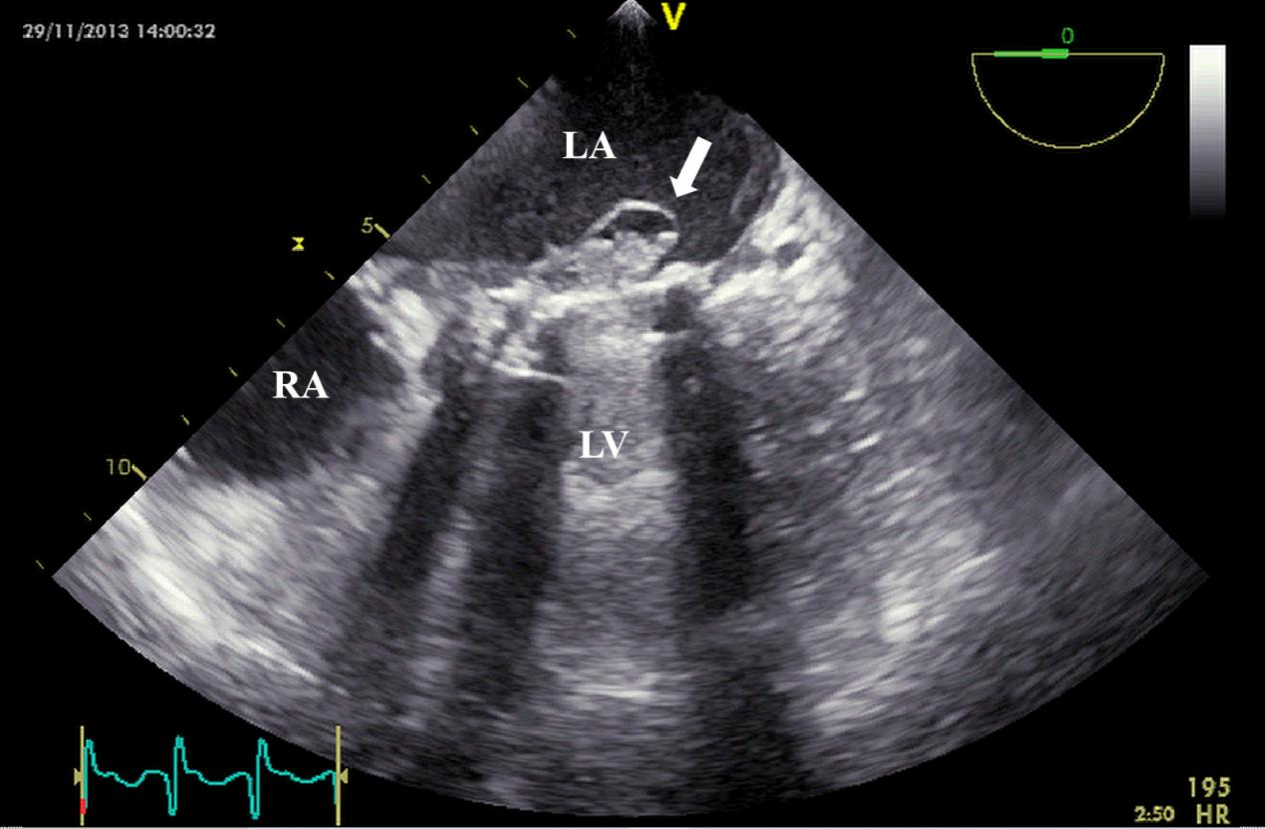
**TEE detection of mechanical valve abscess**. TEE mid-esophageal view suggests mitral valve mechanical valve abscess formation (white arrow). TEE, transesophageal echocardiography; RA, right atrium.

Echocardiography is the preferred imaging diagnostic modality for PVE, which can effectively identify valvular lesions and peri-valvular complications, such as abscesses, dehiscence, and paravalvular infections (Figs. 5,6,7). In the early postoperative period, attention should be paid to possible anatomical variations, such as edema-related changes, which may mimic pathological findings [23]. TEE is superior to TTE in terms of sensitivity (91% vs 65%), and its advantage mainly lies in being less susceptible to the interference of prosthetic valve artifacts. Among patients with suspected PVE, the NPV of TTE and TEE were both relatively high, ranging from 86% to 94% [9]. Furthermore, compared with two-dimensional (2D) TEE, 3D TEE can provide more comprehensive images of cardiac structures and has higher diagnostic value in the analysis of valvular lesions [46].

CCT performs better in identifying prosthetic valve artifact lesions compared to TEE. A clinical study has shown that after incorporating CCT into routine diagnostic evaluation, it can change the treatment decisions of 21% of cases by more accurately identifying perivalvular abscesses and valve dehiscence [47]. Whole-body CT can also detect extracardiac complications, which is helpful for comprehensive diagnosis [20]. Therefore, CT, as a powerful supplement to the preliminary assessment of echocardiography, provides more comprehensive information for a thorough assessment.

Unlike MRI, ^18^F-FDG PET/CT has significant advantages in the diagnosis of IE, especially PVE. Recent meta-analyses have confirmed its diagnostic efficacy. The sensitivity of PVE is 86% and the specificity is 84% [8]. The combined use of ^18^F-FDG PET/CT and echocardiography can increase the diagnostic sensitivity of PVE from 65% to 96% [41]. After combining this molecular imaging technique with the modified Duke criteria, the diagnostic sensitivity can be increased from the original 52–70% to 91–97% [48]. Therefore, the 2023 ESC and Duke-ISCVID guidelines have included it as one of the main diagnostic criteria.

Compared with ^18^F-FDG PET/CT, ^99m^Tc-HMPAO-SPECT/CT technology is more accessible but has lower spatial resolution and sensitivity due to its inherent technical limitations [41]. A comparative study showed that ^18^F-FDG PET/CT had a higher sensitivity in IE detection (93% vs. 64%), while ^99m^Tc-HMPAO-SPECT/CT has an advantage in specificity (100% vs. 73%) [10]. The high specificity of ^99m^Tc-HMPAO-SPECT/CT may be related to its utilization of the mechanism of radioactive-labelled leukocyte accumulation, thereby avoiding confounding inflammation that may mimic the presentation of ^18^F-FDG uptake in IE [49]. A meta-analysis showed that the pooled sensitivity of ^99m^Tc-HMPAO-SPECT/CT in diagnosing IE was 86% (95% CI, 77%–92%), and the specificity was 97% (95% CI, 92%–99%) [11]. Therefore, in clinical scenarios where PET/CT technology is unavailable, ^99m^Tc-HMPAO-SPECT/CT is a feasible alternative option [26].

## 4. Cardiac Device Related IE

Cardiac implantable devices are widely used in the treatment of cardiovascular diseases and can effectively prevent the progression of disease. With the extension of average life expectancy and the increase in the aging population, the proportion of cardiac implantable electronic device-related infective endocarditis (CIED-IE) in all cases of IE rose from 1.7% in 2003 to 4.8% in 2017 [50]. This trend has imposed a heavy burden on healthcare systems, highlighting the importance of early diagnosis of CIED-IE.

### 4.1 ICD Related Infection 

Implantable cardioverter defibrillator (ICD) infections are mainly divided into two types: pocket infections and systemic infections. IE is a significant manifestation of systemic infections [51]. Such infections usually originate from the subcutaneous pocket and then spread along the device leads, eventually affecting the intracardiac portions of the lead that come into contact with the right atrium, tricuspid valve, or right ventricle. This process often manifests as the formation of lead vegetations and/or adjacent valvular vegetations. These structures are the key criteria for the diagnosis of CIED-IE.

**Fig. 8.**
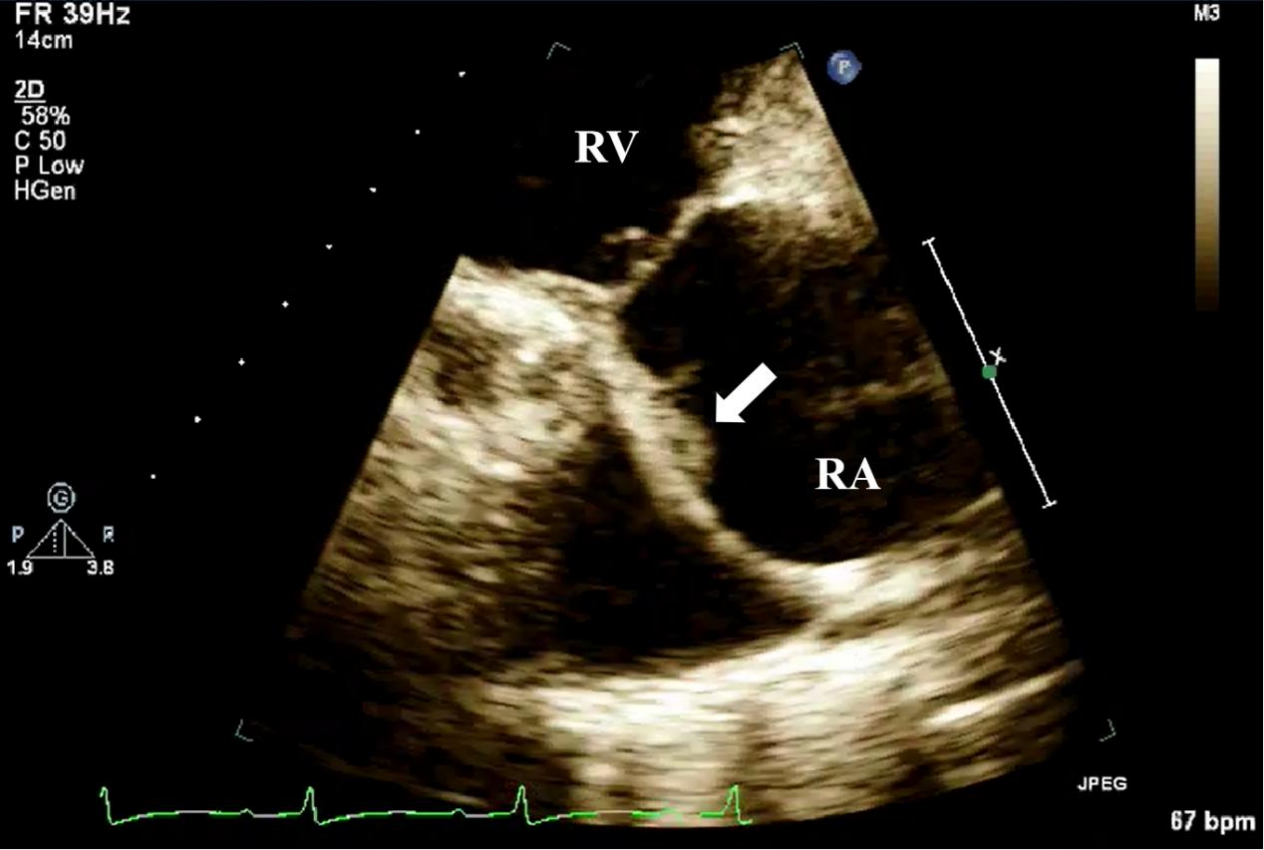
**TTE detection of CIED-IE valve vegetation**. TTE shows moderately echogenic vegetation (white arrow) attached to the pacemaker lead in the right ventricular inflow tract view. CIED-IE, cardiac implantable electronic device-related infective endocarditis; TTE, transthoracic echocardiography; RV, right ventricle.

Echocardiography plays a critical role in the diagnosis of CIED-IE. Studies show that approximately 50% of CIED-IE patients exhibit echocardiographic evidence of valvular vegetations, most commonly on the tricuspid valve (Fig. 8) [51]. TEE is superior to TTE in the diagnostic evaluation of CIED-IE. Tricuspid valves or lead vegetations can be detected in 61% of cases with TEE, but in only 57% with TTE . In addition to the identification of vegetations, TEE can also visualize the leads within the proximal superior vena cava, a region that is often obscured by TTE. In patients with persistently positive blood culture but negative TTE and TEE findings, intracardiac echocardiography (ICE) may be considered for further diagnosis. ICE is widely utilized in the diagnosis of CIED-IE and can more effectively delineate the intracardiac structures. Studies have shown that ICE is more sensitive than TEE in detecting smaller or atypically located lesions [52]. Narducci *et al*. [53] conducted a comparative study of TEE and ICE in 162 CIED-IE patients, and found that the diagnosis of ICE was significantly higher than that of TEE. ICE can be a substitute for TEE especially in patients with esophageal cancer or those in whom cardiac structures need to be evaluated prior to lead extraction. Although ICE has high spatial and temporal resolution, its main limitation lies in its invasiveness. Due to the risk of complications, it is recommended to give priority to ICE in patients with who already have established vascular access [54].

^18^F-FDG PET/CT has emerged as a valuable tool for diagnosing inflammation and infectious diseases. Multiple studies have confirmed the effectiveness of this modality in the diagnosis of CIED-IE. A meta-analysis involving 26 studies showed that its sensitivity was 72% and specificity was 83% [8]. In addition to assessing intracardiac infection, this technique can also identify foci of extracardiac infections such as vertebral osteomyelitis, lower extremity abscesses and septic arthritis, thereby serving as a powerful supplementary diagnostic modality [55]. The 2023 ESC and Duke-ISCVID guidelines have formally integrated ^18^F-FDG PET/CT into the imaging diagnostic framework of IE and listed its metabolic imaging signatures (such as abnormal uptake of device leads or valvular structures) as the major diagnostic criteria for IE. For patients suspected of CIED-IE, TTE and TEE remain the first-line imaging modalities. When the TEE result is inconclusive or fails to confirm the diagnosis, it is recommended to use ^18^F-FDG PET/CT as a supplementary imaging method to improve the diagnostic accuracy and locate the focus of infection.

^99m^Tc-HMPAO-SPECT/CT demonstrates unique clinical value in the diagnosis and risk stratification of CIED-IE. The diagnostic accuracy of this modality is relatively high, with a reported sensitivity of 84%, specificity of 88%, NPV of 93%, and PPV of 74% [12]. Its exceptional NPV helps to effectively exclude CIED-IE and reduce diagnostic uncertainty. After being included in the modified Duke criteria, the proportion of the “possible CIED-IE” classifications decreased from 49.5% to 37%, significantly improving the diagnostic accuracy [12]. In addition to its diagnostic value, this technique also has prognostic significance: its positive results correlate with elevated mortality during hospitalization, increased complications, and an increased likelihood of definite removal of the cardiac implanted device [56].

### 4.2 Ventricular Assist Device Infection

Ventricular assist devices (VAD) are used as a bridge to recovery in patients with heart failure, can serve as supportive treatment during heart transplantation, or as destination therapy for end-stage heart failure. VAD therapy can be used as a left ventricular assist device (LVAD), right ventricular assist device (RVAD), and biventricular assist device (BiVAD). Currently, LVAD-related infection is the most common and serious complication, affecting approximately 20–40% of patients, and often develops into sepsis within 1–2 years after implantation [57].

Recent research has shown that the nuclear imaging technique plays an important role in this field. A small non-randomized study reported that WBC SPECT/CT demonstrated outstanding accuracy in diagnosing LVAD infection [58]. Similarly, ^18^F-FDG PET/CT shows a high sensitivity (82–97%) and variable specificity (24–99%) in infection detection [59]. ^18^F-FDG PET/CT is also of great significance in guiding treatment strategies. Sohns *et al*. [60] demonstrated that patients who underwent surgical repair surgery within 3 months after PET/CT had significantly fewer hospitalizations during subsequent follow-up than those who received delayed intervention.

However, current evidence in VAD-related infection is predominantly from retrospective studies, and there is still a lack of multicenter randomized prospective trials to verify its diagnostic and prognostic value. Therefore, it is necessary to carry out large-scale and prospective clinical trials to further establish the role of this type of imaging technology in the diagnosis and management of VAD infection.

## 5. Transcatheter Prosthetic Valve Endocarditis

With the wide application of transcatheter aortic valve implantation (TAVI), also known as transcatheter aortic valve replacement (TAVR), the incidence of TAVI-related IE (TAVI-IE) has also significantly increased. TAVI delivers a bioprosthetic valve via a catheter to replace the diseased aortic valve. Compared with open, invasive surgical techniques, TAVI is less invasive, results in a shorter postoperative recovery time, and has fewer postoperative complications [26].

Although echocardiography remains the first-line imaging modality for suspected TAVI-IE, its diagnostic efficacy is significantly inferior to that of NVE or PVE. The main limitations include the lack of surgical anatomical landmarks (e.g., sewing rings) and acoustic shadowing caused by the metallic stent. Even with TEE, smaller vegetations cannot be detected in 38%–60% of cases [61]. ^18^F-FDG PET/CT and CTA have been proven to be important ancillary methods, which can identify abnormal metabolic activity or periprosthetic lesions, thereby reclassifying and diagnosing PVE in 33% of cases [62].

Similar to TAVI, transcatheter pulmonary valve implantation (TPVI) is a surgical procedure in which a prosthetic valve is implanted in the pulmonary valve via a catheter without the need for a thoracotomy. It is often used to treat patients with pulmonary valve insufficiency or structural abnormalities. The diagnosis of IE after TPVI is complex. When the results of TTE or TEE are negative, but there is a high clinical suspicion of infection, integrating ICE and ^18^F-FDG PET/CT has important diagnostic value, especially in identifying perivalvular abscesses or distant infections [63]. The main pathogens of IE after TPVI are *Staphylococcus aureus* and *streptococci* (e.g., *Streptococcus mitis*, *S. sanguinis*), suggesting that the infection mostly originates from hematogenous dissemination in the mucosal areas or oral cavity.

## 6. Conclusions

Multimodality imaging techniques play an important role throughout the entire process of diagnosis and treatment of IE, including the diagnosis, risk assessment, treatment monitoring and prognosis. Echocardiography is the preferred primary screening method and is applicable to most suspected cases. CT performs superiorly in identifying perivalvular abscesses and pseudoaneurysms; MRI is extremely sensitive to systemic embolism and changes in myocarditis. ^18^F-FDG PET/CT can more accurately locate the foci of infection in prosthetic valves and cardiac implant devices. With the continuous advancement of medical technology, the imaging diagnosis of IE is moving towards a new stage of multimodality integration. Future research will focus on developing specific molecular tracers targeting bacterial metabolic pathways or cell membrane components, such as ^11^C-para-aminobenzoic acid and 2-[^18^F]F-p-aminobenzoic acid PET tracers, which have been shown to have selective uptake of pathogenic microorganisms and are expected to be more frequently used as diagnostic tools in IE [64, 65]. By integrating imaging results with clinical parameters, characteristic lesions can be quickly identified with the help of machine learning, and models for predicting disease progression and treatment outcomes can be constructed. This trend will promote the development and evaluation of new IE imaging techniques, resulting in more accurate diagnosis of IE, which will contribute to improved patient outcomes [66].

## References

[1] Cahill TJ, Prendergast BD (2016). Infective endocarditis. *Lancet*.

[2] Nesseler N, Launey Y, Mallédant Y (2013). Infective endocarditis. *The New England Journal of Medicine*.

[3] Momtazmanesh S, Saeedi Moghaddam S, Malakan Rad E, Azadnajafabad S, Ebrahimi N, Mohammadi E (2022). Global, regional, and national burden and quality of care index of endocarditis: the global burden of disease study 1990-2019. *European Journal of Preventive Cardiology*.

[4] Fournier JB, Testa EJ (2019). Nonbacterial Thrombotic Endocarditis. *The New England Journal of Medicine*.

[5] Thuny F, Habib G, Le Dolley Y, Canault M, Casalta JP, Verdier M (2011). Circulating matrix metalloproteinases in infective endocarditis: a possible marker of the embolic risk. *PloS One*.

[6] Bonzi M, Cernuschi G, Solbiati M, Giusti G, Montano N, Ceriani E (2018). Diagnostic accuracy of transthoracic echocardiography to identify native valve infective endocarditis: a systematic review and meta-analysis. *Internal and Emergency Medicine*.

[7] Jain V, Wang TKM, Bansal A, Farwati M, Gad M, Montane B (2021). Diagnostic performance of cardiac computed tomography versus transesophageal echocardiography in infective endocarditis: A contemporary comparative meta-analysis. *Journal of Cardiovascular Computed Tomography*.

[8] Wang TKM, Sánchez-Nadales A, Igbinomwanhia E, Cremer P, Griffin B, Xu B (2020). Diagnosis of Infective Endocarditis by Subtype Using 18F-Fluorodeoxyglucose Positron Emission Tomography/Computed Tomography: A Contemporary Meta-Analysis. *Circulation. Cardiovascular Imaging*.

[9] Ivanovic B, Trifunovic D, Matic S, Petrovic J, Sacic D, Tadic M (2019). Prosthetic valve endocarditis - A trouble or a challenge?. *Journal of Cardiology*.

[10] Rouzet F, Chequer R, Benali K, Lepage L, Ghodbane W, Duval X (2014). Respective performance of 18F-FDG PET and radiolabeled leukocyte scintigraphy for the diagnosis of prosthetic valve endocarditis. *Journal of Nuclear Medicine*.

[11] Juneau D, Golfam M, Hazra S, Erthal F, Zuckier LS, Bernick J (2018). Molecular Imaging for the diagnosis of infective endocarditis: A systematic literature review and meta-analysis. *International Journal of Cardiology*.

[12] Holcman K, Małecka B, Rubiś P, Ząbek A, Szot W, Boczar K (2020). The role of 99mTc-HMPAO-labelled white blood cell scintigraphy in the diagnosis of cardiac device-related infective endocarditis. *European Heart Journal. Cardiovascular Imaging*.

[13] Chambers HF, Bayer AS (2020). Native-Valve Infective Endocarditis. *The New England Journal of Medicine*.

[14] Barbieri A, Cecchi E, Bursi F, Mantovani F (2023). Is Infectious Endocarditis Evolving into a Time-Dependent Diagnosis in the Contemporary Epidemiological Era? Emphasis on the Role of Echocardiography as a First-Line Diagnostic Approach. *Reviews in Cardiovascular Medicine*.

[15] Castillo Almeida NE, Gurram P, Esquer Garrigos Z, Mahmood M, Baddour LM, Sohail MR (2020). Diagnostic imaging in infective endocarditis: a contemporary perspective. *Expert Review of Anti-infective Therapy*.

[16] Gomes A, Glaudemans AWJM, Touw DJ, van Melle JP, Willems TP, Maass AH (2017). Diagnostic value of imaging in infective endocarditis: a systematic review. *The Lancet. Infectious Diseases*.

[17] Mir T, Uddin M, Qureshi WT, Regmi N, Tleyjeh IM, Saydain G (2022). Predictors of Complications Secondary to Infective Endocarditis and Their Associated Outcomes: A Large Cohort Study from the National Emergency Database (2016-2018). *Infectious Diseases and Therapy*.

[18] Pericàs JM, Hernández-Meneses M, Muñoz P, Martínez-Sellés M, Álvarez-Uria A, de Alarcón A (2021). Characteristics and Outcome of Acute Heart Failure in Infective Endocarditis: Focus on Cardiogenic Shock. *Clinical Infectious Diseases*.

[19] Khalique OK, Veillet-Chowdhury M, Choi AD, Feuchtner G, Lopez-Mattei J (2021). Cardiac computed tomography in the contemporary evaluation of infective endocarditis. *Journal of Cardiovascular Computed Tomography*.

[20] Palmisano A, Bruno E, Vignale D, Bognoni L, Ascione R, Ingallina G (2025). Comprehensive CT study to assess local and systemic involvement in patients with infective endocarditis: experience from a multidisciplinary team of a tertiary referral center. *La Radiologia Medica*.

[21] Petkovic A, Menkovic N, Petrovic O, Bilbija I, Radovanovic NN, Stanisavljevic D (2023). The Role of Echocardiography and Cardiac Computed Tomography in Diagnosis of Infective Endocarditis. *Journal of Clinical Medicine*.

[22] Oliveira M, Guittet L, Hamon M, Hamon M (2020). Comparative Value of Cardiac CT and Transesophageal Echocardiography in Infective Endocarditis: A Systematic Review and Meta-Analysis. *Radiology. Cardiothoracic Imaging*.

[23] Horgan SJ, Mediratta A, Gillam LD (2020). Cardiovascular Imaging in Infective Endocarditis: A Multimodality Approach. *Circulation. Cardiovascular Imaging*.

[24] De Stasio V, Delahaye F, Moreau-Triby C, Pozzi M, Si-Mohamed S, Douek P (2021). Integrated imaging evaluation in infective endocarditis: A pictorial essay on clinical cases of extracardiac complications. *International Journal of Infectious Diseases*.

[25] Duval X, Iung B (2017). Extracardiac Imaging of Infective Endocarditis. *Current Infectious Disease Reports*.

[26] Delgado V, Ajmone Marsan N, de Waha S, Bonaros N, Brida M, Burri H (2023). 2023 ESC Guidelines for the management of endocarditis. *European Heart Journal*.

[27] Imazio M, Pivetta E, Palacio Restrepo S, Sormani P, Pedrotti P, Quarta G (2020). Usefulness of Cardiac Magnetic Resonance for Recurrent Pericarditis. *The American Journal of Cardiology*.

[28] Fowler VG, Durack DT, Selton-Suty C, Athan E, Bayer AS, Chamis AL (2023). The 2023 Duke-International Society for Cardiovascular Infectious Diseases Criteria for Infective Endocarditis: Updating the Modified Duke Criteria. *Clinical Infectious Diseases*.

[29] Vitali P, Savoldi F, Segati F, Melazzini L, Zanardo M, Fedeli MP (2022). MRI versus CT in the detection of brain lesions in patients with infective endocarditis before or after cardiac surgery. *Neuroradiology*.

[30] Ahn Y, Joo L, Suh CH, Kim S, Shim WH, Kim SJ (2022). Impact of Brain MRI on the Diagnosis of Infective Endocarditis and Treatment Decisions: Systematic Review and Meta-Analysis. *AJR. American Journal of Roentgenology*.

[31] Papadimitriou-Olivgeris M, Guery B, Ianculescu N, Dunet V, Messaoudi Y, Pistocchi S (2023). Role of Cerebral Imaging on Diagnosis and Management in Patients With Suspected Infective Endocarditis. *Clinical Infectious Diseases*.

[32] Oh JK, Jung J, Lee SA, Lee S, Lee EJ, Chang E (2023). Impact of routine brain imaging on the prognosis of patients with left-sided valve infective endocarditis without neurological manifestations. *International Journal of Cardiology*.

[33] Chakraborty T, Scharf E, DeSimone D, El Rafei A, Brinjikji W, Baddour LM (2019). Variable Significance of Brain MRI Findings in Infective Endocarditis and Its Effect on Surgical Decisions. *Mayo Clinic Proceedings*.

[34] Rahmim A, Zaidi H (2008). PET versus SPECT: strengths, limitations and challenges. *Nuclear Medicine Communications*.

[35] de Camargo RA, Sommer Bitencourt M, Meneghetti JC, Soares J, Gonçalves LFT, Buchpiguel CA (2020). The Role of 18F-Fluorodeoxyglucose Positron Emission Tomography/Computed Tomography in the Diagnosis of Left-sided Endocarditis: Native vs Prosthetic Valves Endocarditis. *Clinical Infectious Diseases*.

[36] Duval X, Le Moing V, Tubiana S, Esposito-Farèse M, Ilic-Habensus E, Leclercq F (2021). Impact of Systematic Whole-body 18F-Fluorodeoxyglucose PET/CT on the Management of Patients Suspected of Infective Endocarditis: The Prospective Multicenter TEPvENDO Study. *Clinical Infectious Diseases*.

[37] Abikhzer G, Martineau P, Gregoire J, Finnerty V, Harel F, Pelletier-Galarneau M (2022). [^18^F]FDG-PET CT for the evaluation of native valve endocarditis. *Journal of Nuclear Cardiology*.

[38] Gazzilli M, Albano D, Lucchini S, Peli A, Cerudelli E, Bertagna F (2022). New criteria for the diagnosis of infective endocarditis using 18F-FDG PET/CT imaging. *Journal of Nuclear Cardiology*.

[39] Philip M, Delcourt S, Mancini J, Tessonnier L, Cammilleri S, Arregle F (2021). ^18^F-fluorodeoxyglucose positron emission tomography/computed tomography for the diagnosis of native valve infective endocarditis: A prospective study. *Archives of Cardiovascular Diseases*.

[40] Granados U, Fuster D, Pericas JM, Llopis JL, Ninot S, Quintana E (2016). Diagnostic Accuracy of 18F-FDG PET/CT in Infective Endocarditis and Implantable Cardiac Electronic Device Infection: A Cross-Sectional Study. *Journal of Nuclear Medicine*.

[41] Swart LE, Gomes A, Scholtens AM, Sinha B, Tanis W, Lam MGEH (2018). Improving the Diagnostic Performance of 18F-Fluorodeoxyglucose Positron-Emission Tomography/Computed Tomography in Prosthetic Heart Valve Endocarditis. *Circulation*.

[42] Erba PA, Conti U, Lazzeri E, Sollini M, Doria R, De Tommasi SM (2012). Added value of 99mTc-HMPAO-labeled leukocyte SPECT/CT in the characterization and management of patients with infectious endocarditis. *Journal of Nuclear Medicine*.

[43] Holcman K, Szot W, Rubiś P, Leśniak-Sobelga A, Hlawaty M, Wiśniowska-Śmiałek S (2019). 99mTc-HMPAO-labeled leukocyte SPECT/CT and transthoracic echocardiography diagnostic value in infective endocarditis. *The International Journal of Cardiovascular Imaging*.

[44] Holcman K, Rubiś P, Ząbek A, Boczar K, Podolec P, Kostkiewicz M (2023). Advances in Molecular Imaging in Infective Endocarditis. *Vaccines*.

[45] Holcman K, Rubiś P, Ćmiel B, Ząbek A, Boczar K, Szot W (2023). To what extent does prior antimicrobial therapy affect the diagnostic performance of radiolabeled leukocyte scintigraphy in infective endocarditis?. *Journal of Nuclear Cardiology*.

[46] Tomoaia R, Beyer RȘ, Dădârlat-Pop A, Șerban AM, Pop D, Zdrenghea D (2024). Novel 3D versus traditional transesophageal echocardiography techniques: Defining differences in the diagnosis of infective endocarditis. *European Journal of Clinical Investigation*.

[47] Harding D, Prendergast B (2018). Advanced imaging improves the diagnosis of infective endocarditis. *F1000Research*.

[48] Pizzi MN, Roque A, Fernández-Hidalgo N, Cuéllar-Calabria H, Ferreira-González I, Gonzàlez-Alujas MT (2015). Improving the Diagnosis of Infective Endocarditis in Prosthetic Valves and Intracardiac Devices With 18F-Fluordeoxyglucose Positron Emission Tomography/Computed Tomography Angiography: Initial Results at an Infective Endocarditis Referral Center. *Circulation*.

[49] Lee JC, Wee YS, Horvath RL (2020). Nuclear imaging in the diagnosis of infective endocarditis. *Journal of Nuclear Cardiology*.

[50] Khaloo P, Uzomah UA, Shaqdan A, Ledesma PA, Galvin J, Ptaszek LM (2022). Outcomes of Patients Hospitalized With Cardiovascular Implantable Electronic Device-Related Infective Endocarditis, Prosthetic Valve Endocarditis, and Native Valve Endocarditis: A Nationwide Study, 2003 to 2017. *Journal of the American Heart Association*.

[51] Sohail MR, Uslan DZ, Khan AH, Friedman PA, Hayes DL, Wilson WR (2008). Infective endocarditis complicating permanent pacemaker and implantable cardioverter-defibrillator infection. *Mayo Clinic Proceedings*.

[52] Baddour LM, Epstein AE, Erickson CC, Knight BP, Levison ME, Lockhart PB (2010). Update on cardiovascular implantable electronic device infections and their management: a scientific statement from the American Heart Association. *Circulation*.

[53] Narducci ML, Pelargonio G, Russo E, Marinaccio L, Di Monaco A, Perna F (2013). Usefulness of intracardiac echocardiography for the diagnosis of cardiovascular implantable electronic device-related endocarditis. *Journal of the American College of Cardiology*.

[54] Sanchez-Nadales A, Cedeño J, Sonnino A, Sarkar A, Igbinomwanhia E, Asher CR (2023). Utility of Intracardiac Echocardiography for Infective Endocarditis and Cardiovascular Device-Related Endocarditis: A Contemporary Systematic Review. *Current Problems in Cardiology*.

[55] Hohmann C, Michels G, Schmidt M, Pfister R, Mader N, Ohler M (2019). Diagnostic challenges in infective endocarditis: is PET/CT the solution?. *Infection*.

[56] Holcman K, Rubiś P, Ząbek A, Ćmiel B, Szot W, Boczar K (2020). The Prognostic Value of 99mTc-HMPAO-Labeled Leucocyte SPECT/CT in Cardiac Device-Related Infective Endocarditis. *JACC. Cardiovascular Imaging*.

[57] Kim J, Feller ED, Chen W, Liang Y, Dilsizian V (2019). FDG PET/CT for Early Detection and Localization of Left Ventricular Assist Device Infection: Impact on Patient Management and Outcome. *JACC. Cardiovascular Imaging*.

[58] Litzler PY, Manrique A, Etienne M, Salles A, Edet-Sanson A, Vera P (2010). Leukocyte SPECT/CT for detecting infection of left-ventricular-assist devices: preliminary results. *Journal of Nuclear Medicine*.

[59] Tam MC, Patel VN, Weinberg RL, Hulten EA, Aaronson KD, Pagani FD (2020). Diagnostic Accuracy of FDG PET/CT in Suspected LVAD Infections: A Case Series, Systematic Review, and Meta-Analysis. *JACC. Cardiovascular Imaging*.

[60] Sohns JM, Bavendiek U, Ross TL, Bengel FM (2017). Targeting Cardiovascular Implant Infection: Multimodality and Molecular Imaging. *Circulation. Cardiovascular Imaging*.

[61] Bjursten H, Rasmussen M, Nozohoor S, Götberg M, Olaison L, Rück A (2019). Infective endocarditis after transcatheter aortic valve implantation: a nationwide study. *European Heart Journal*.

[62] Wahadat AR, Tanis W, Swart LE, Scholtens A, Krestin GP, van Mieghem NMDA (2021). Added value of 18F-FDG-PET/CT and cardiac CTA in suspected transcatheter aortic valve endocarditis. *Journal of Nuclear Cardiology*.

[63] Venet M, Jalal Z, Ly R, Malekzadeh-Milani S, Hascoët S, Fournier E (2022). Diagnostic Value of 18F-Fluorodeoxyglucose Positron Emission Tomography Computed Tomography in Prosthetic Pulmonary Valve Infective Endocarditis. *JACC. Cardiovascular Imaging*.

[64] Mutch CA, Ordonez AA, Qin H, Parker M, Bambarger LE, Villanueva-Meyer JE (2018). 11C]Para-Aminobenzoic Acid: A Positron Emission Tomography Tracer Targeting Bacteria-Specific Metabolism. *ACS Infectious Diseases*.

[65] Schulte J, Maurer A, Domogalla LC, Steinacker N, Wadle C, Kinzler J (2025). 2-[18F]F-p-Aminobenzoic Acid Specifically Detects Infective Endocarditis in Positron Emission Tomography. *The Journal of Infectious Diseases*.

[66] M Odat R, Marsool Marsool MD, Nguyen D, Idrees M, Hussein AM, Ghabally M (2024). Presurgery and postsurgery: advancements in artificial intelligence and machine learning models for enhancing patient management in infective endocarditis. *International Journal of Surgery*.

